# ASO Author Reflections: Reassessing Positive Margins in Breast-Conserving Surgery: The Role of Histology and Oncoplastic Surgery

**DOI:** 10.1245/s10434-025-17542-z

**Published:** 2025-06-06

**Authors:** Kayla M. Switalla, Rita A. Mukhtar

**Affiliations:** 1 University of Minnesota Medical School, Minneapolis, USA; 2 Department of Surgery, University of California San Francisco, San Francisco, USA; 3 UCSF Helen Diller Family Comprehensive Cancer Center, Department of Surgery, San Francisco, USA

## Past

The evolution of breast cancer surgery has increasingly emphasized not only oncologic safety but also patient-centered outcomes, such as aesthetics and quality of life. Oncoplastic breast-conserving surgery (BCS) emerged from this shift, combining oncologic resection with plastic surgery techniques to allow wider excisions without compromising cosmetic results.^1^ By allowing for larger resections, oncoplastic BCS may also enhance oncologic outcomes by lowering the risk of positive margins. Several studies support this benefit of reduced positive margin rates overall after oncoplastic BCS; yet, few have explored this outcome specifically in patients with invasive lobular carcinoma (ILC), a histologic subtype known for its insidious growth and complex surgical planning.^1,2^ Historically, ILC, compared with IDC, has been associated with higher rates of margin positivity following standard BCS, raising questions about whether oncoplastic approaches may offer a distinct advantage in this population.^3^ Despite its increasing adoption, the impact of oncoplastic BCS on surgical margins, particularly for ILC, remains poorly defined, highlighting the need for focused investigation.

## Present

Currently, patients with ILC still face higher rates of positive margins compared with patients with IDC after standard BCS.^4^ Indeed, in our meta-analysis and systematic review of more than 700 patients with ILC, the positive margin rate for ILC patients undergoing oncoplastic BCS remained substantial at 31% (95% confidence interval [CI] 21–40%).^5^ Notably, this risk remained significantly elevated compared with IDC even when utilizing oncoplastic approaches (relative risk [RR] 3.4, 95% CI 1.5–7.4).^5^

However, an important finding emerged when investigating subpopulations of ILC patients. Interestingly, for ILC patients with larger tumors, oncoplastic BCS demonstrated a statistically significant advantage in lowering positive margin risk compared to standard BCS.^5^ Because a large proportion of ILC patients in our review underwent shave margins in addition to oncoplastic surgery, this suggests that for patients with larger tumors, shave margins alone may not suffice to reduce the risk of positive margins, making oncoplastic surgery particularly beneficial.

## Future

Taken together, these findings highlight the benefit of oncoplastic BCS in reducing positive margin rates particularly among ILC patients with large tumors. However, despite its advantages, a persistent disparity in margin positivity between ILC and IDC remains. This underscores the critical need for improved preoperative imaging techniques for ILC and further evaluation of neoadjuvant treatment approaches for ILC. In the meantime, oncoplastic approaches appear to benefit patients with ILC, especially for those with larger tumors, and will likely comprise an important part of the strategy to improve surgical outcomes in this high-risk population.
